# Incomplete Assembly of the Dystrophin-Associated Protein Complex in 2D and 3D-Cultured Human Induced Pluripotent Stem Cell-Derived Cardiomyocytes

**DOI:** 10.3389/fcell.2021.737840

**Published:** 2021-11-04

**Authors:** Guillaume Gilbert, Chandan Kadur Nagaraju, Robin Duelen, Matthew Amoni, Pierre Bobin, Thomas Eschenhagen, H. Llewelyn Roderick, Maurilio Sampaolesi, Karin R. Sipido

**Affiliations:** ^1^Laboratory of Experimental Cardiology, Department of Cardiovascular Sciences, KU Leuven, Leuven, Belgium; ^2^Laboratory of Translational Cardiomyology, Department of Development and Regeneration, Stem Cell Institute, KU Leuven, Leuven, Belgium; ^3^Institute of Experimental Pharmacology and Toxicology, University Medical Center Hamburg-Eppendorf, Hamburg, Germany; ^4^German Centre for Cardiovascular Research (DZHK), Partner Site Hamburg/Kiel/Lübeck, Hamburg, Germany

**Keywords:** dystrophin-associated glycoprotein complex, human induced pluripotent stem cells, hiPSC-derived cardiomyocytes, sarcoglycanopathy, hiPSC cardiomyocyte maturation, Duchenne muscular dystrophy, cardiomyopathy

## Abstract

Human induced pluripotent stem cells derived cardiomyocytes (hiPSC-CM) are increasingly used to study genetic diseases on a human background. However, the lack of a fully mature adult cardiomyocyte phenotype of hiPSC-CM may be limiting the scope of these studies. Muscular dystrophies and concomitant cardiomyopathies result from mutations in genes encoding proteins of the dystrophin-associated protein complex (DAPC), which is a multi-protein membrane-spanning complex. We examined the expression of DAPC components in hiPSC-CM, which underwent maturation in 2D and 3D culture protocols. The results were compared with human adult cardiac tissue and isolated cardiomyocytes. We found that similarly to adult cardiomyocytes, hiPSC-CM express dystrophin, in line with previous studies on Duchenne’s disease. β-dystroglycan was also expressed, but, contrary to findings in adult cardiomyocytes, none of the sarcoglycans nor α-dystroglycan were, despite the presence of their mRNA. In conclusion, despite the robust expression of dystrophin, the absence of several other DAPC protein components cautions for reliance on commonly used protocols for hiPSC-CM maturation for functional assessment of the complete DAPC.

## Introduction

**FIGURE 1:**
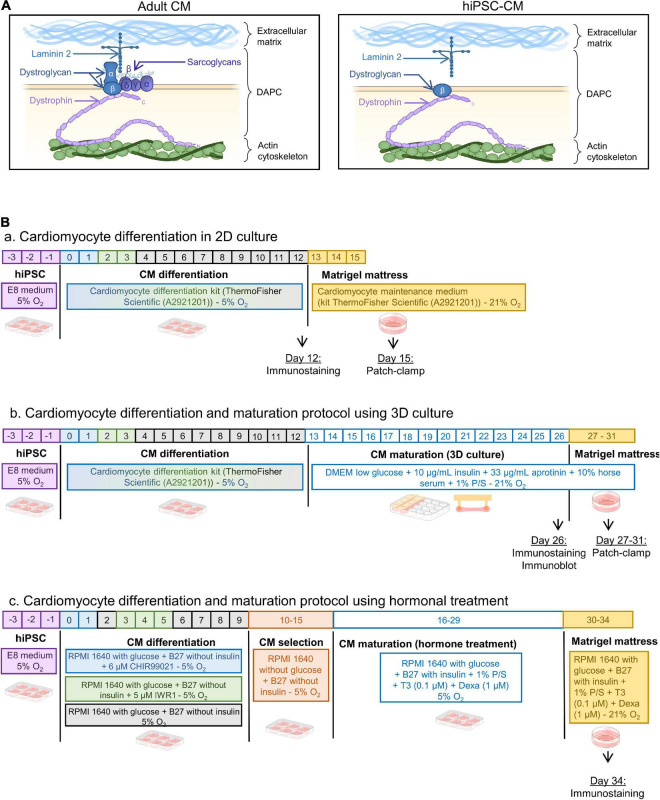
**(A)** Schematic of the DAPC composition in adult vs. hiPSC-CM. Laminin was present in the hiPSC-CM preparations (See Supplementary Figures 1,2). **(B)** Differentiation protocols of hiPSC-CM: panel **(a)** in 2D monolayer culture, panel **(b)** in 3D engineered heart tissues, and panel **(c)** with thyroid hormone and glucocorticoid treatment followed by Matrigel mattresses.

Muscular dystrophies are genetically inherited degenerative disorders with a progressive impairment of skeletal, respiratory, and cardiac function (Mercuri et al., 2019). The most prevalent muscular dystrophies involve proteins from the dystrophin-associated protein complex (DAPC) with dystrophin, sarcoglycans, dystroglycans, and laminin as core components (Figure 1A, left). The DAPC has a mechanical and signaling role in muscle cells, providing a link between the extracellular matrix and the intracellular cytoskeleton (Cohn and Campbell, 2000; Ozawa, 2010). Studies in animal models for muscular dystrophies provided insights into the mechanistic pathways leading to the development of cardiomyopathy (loss of membrane integrity, increase in cell permeability, cardiomyocyte cell death, and replacement fibrosis) (Ikeda et al., 2000; Heydemann et al., 2001; Lapidos et al., 2004; Fraysse et al., 2010; Law et al., 2020). However, because of the unavailability of cardiac biopsies from those patients, there remains a knowledge gap in the understanding of the cellular mechanisms underlying cardiomyopathy in humans, hampering clinical translation. To overcome this limitation, human induced pluripotent stem cell-derived cardiomyocytes (hiPSC-CM) are increasingly used as a model. A leading example is Duchenne muscular dystrophy, resulting from loss of dystrophin, which has been studied extensively with important translational insights (Long et al., 2018; Kamdar et al., 2020; Moretti et al., 2020; Pioner et al., 2020; Mekies et al., 2021). Notwithstanding the advantages of using human cells, a general limitation of the approach is that hiPSC-CMs lack several features of adult cardiomyocytes, presumably due to incomplete maturation, resulting in a fetal or neonatal phenotype (Guo and Pu, 2020; Karbassi et al., 2020). Multiple strategies have been presented to promote hiPSC-CM maturation (reviewed in Ahmed et al., 2020). These rely on hormonal treatment, imposing load and pacing, or a 3D environment. HiPSC-CM generated *via* some of these methods have been used in the study of Duchenne muscular dystrophy (Long et al., 2018; Pioner et al., 2020), yet it has also been suggested that dystrophin is needed for hiPSC-CM maturation (Pioner et al., 2020), and presently it is unknown whether the DAPC in hiPSC-CM forms a complete functional complex.

The present study examines the presence of the DAPC in hiPSC-CM, using maturation protocols that are accessible and commonly used (Figure 1B). The first is the well-established technique to create a small-engineered heart tissue by culturing the cells in a 3D microenvironment. Cells are embedded in a fibrin/Matrigel hydrogel connected to silicone posts that will exert a tension force, mimicking the preload tension on a muscle fiber (Jackman et al., 2016; Breckwoldt et al., 2017; Tiburcy et al., 2017; Leonard et al., 2018; Goldfracht et al., 2020). The second maturation method is a protocol that has been shown to structurally improve iPSC-CM membrane with the presence of transverse tubules, an important hallmark of cardiomyocyte maturity, by stimulating 2D cultured hiPSC-CM with thyroid hormones and glucocorticoids (Parikh et al., 2017; Huang et al., 2020). The data are compared with the hiPSC-CM differentiated in 2D without an intensified maturation protocol, and with adult human cardiac tissue.

## Method

### Human Induced Pluripotent Stem Cell Lines

We used a commercial hiPSC line from ThermoFisher Scientific (A18945—lot 1793435) and three additional non-commercial hiPSC lines, one derived within the Stem Cell Institute at KU Leuven, (HC1) and two elaborated at the University Medical Center, Hamburg Lab (ERC001 and ERC018).

### Cardiomyocyte Differentiation and Maturation Protocol Using 3D Culture of Human Induced Pluripotent Stem Cell-Derived Cardiomyocytes

HiPSC were differentiated in 2D monolayers using the cardiomyocyte differentiation kit from ThermoFisher Scientific (A2921201) (Figure 1B, panel a). Twelve days post-differentiation, hiPSC-CM were placed in a fibrin and Matrigel based 3D environment using the system developed in the Eschenhagen group (Schaaf et al., 2014; Breckwoldt et al., 2017), modified in a mixture as described here (Jackman et al., 2016). Briefly, three wells of 2D differentiated cells were detached using collagenase A at 1 U/ml (Merk—10103586001) and pooled to prepare 3D constructs in a mixture of Matrigel (10% final volume) (Corning—354234), fibrinogen (20% final volume at 2 mg/ml) (Merk—341576), and thrombin (2% final volume at 1 U/ml) (Enzyme Research Laboratories—HT 1002a). The culture medium composition was as follows: DMEM low glucose (ThermoFisher Scientific—31885023), 10% horse serum (ThermoFisher Scientific—26050088), 1% penicillin/streptomycin, 10 μg/ml human insulin (Sigma-Aldrich—I9278-5ML), and 33 μg/ml aprotinin (Carl Roth—A162.3). The medium was replaced every 2 days for 14 days (day 26 post initial differentiation) (Figure 1B, panel b).

### Cardiomyocyte Differentiation and Maturation Protocol Using Chemical Treatment

The protocol used here was the same as previously described (Parikh et al., 2017). Briefly, hiPSC were differentiated in RPMI 1640 medium containing glucose (ThermoFisher Scientific—11875093) supplemented with B27 without insulin (ThermoFisher Scientific—A1895601), using 6 μM CHIR99021 (Merk—SML1046) on day 1 followed by 5 μM IWR-1 (Merk—I0161) on day 3. From days 10 to 16, glucose was removed from the medium to perform a metabolic selection and cardiomyocyte enrichment (ThermoFisher Scientific—11879020). Cells were then treated with 0.1 μM triiodo-L-thyronine hormone (Merk—T2877), 1 μM dexamethasone (Cayman—11015) in RPMI 1640 with glucose (ThermoFisher Scientific—11875093) supplemented with B27 (ThermoFisher Scientific—17504044), and 1% penicillin/streptomycin. On day 30, the 2D monolayer of hiPSC-CM was dissociated using TrypLE Express (ThermoFisher Scientific—12605010) and seeded onto Matrigel mattresses (Corning—354234) for 4 days until experiments (Figure 1B, panel c).

### Proteasome Inhibition Test

Three-dimensional cultured hiPSC-CM were incubated in the 37°C incubator with 10 μM of MG-132 (Merk—474787) in the culture medium for 8 h. After 8 h, the 3D hiPSC-CM were snap frozen in liquid nitrogen for further analysis by immunoblot. Proteasome inhibition efficiency was confirmed by assessing by immunoblotting for ubiquitinated proteins using a ubiquitin antibody.

### Dissociation of Human Induced Pluripotent Stem Cell From 3D Constructs

To perform electrophysiology experiments, cells grown in 3D were dissociated using 0.4 mg/ml papain (Worthington Biochemical Corporation—LS003118), 0.3 mg/ml collagenase type IV (Worthington Biochemical Corporation—LS004186), 2 mM DL-dithiothreitol (Merk—D0632), 50 μM CaCl_2_, and 1 mg/ml bovine serum albumin (Merk—A2153) in Hank’s balanced salt solution (HBSS) (ThermoFisher Scientific—14170088) for 20 min at 37°C. After centrifugation at 1200 rpm for 5 min, cells were resuspended in the 3D culture medium and seeded on Matrigel mattresses for 1–5 days until experiment, as previously described (Feaster et al., 2015). Briefly, 10 min prior to adding the dissociated cells, thin lines of 20 mm long containing 1 μl of pure Matrigel were poured using a P10 pipet on glass coverslips.

### Adult Human Cardiomyocyte Isolation

Use of tissue from non-used human donor hearts conforms with ethical guidelines, and permission for the study was obtained from the Ethical Committee of UZ Leuven (permit number S58824). Hearts were collected in an ice-cold solution containing (in mM): 130 NaCl, 27 KCl, 6 *N*-2-hydroxyethylpiperazine-*N*-2-ethanesulfonic acid (HEPES), 1.2 MgSO_4_, 1.2 KH_2_PO_4_, and 10 glucose; pH was adjusted to 7.2 with NaOH and transported from the hospital to the laboratory. A coronary artery from a wedge of the left ventricle was cannulated. Then, the wedge was perfused for 30 min with a Ca^2+^ free solution at 37°C bubbled with O_2_ and containing (in mM): 130 NaCl, 5.4 KCl, 6 HEPES, 1.2 MgSO_4_, 1.2 KH_2_PO_4_, and 20 glucose; pH was adjusted to 7.2 with NaOH. After this washing step, the wedge was perfused for 40 min with the same solution containing around 0.4 U/ml of Collagenase A (Merk—10103586001) and 0.1 mg/ml Protease XIV (Merk—P5147). When the tissue appeared digested, it was perfused for 20 min with a low Ca^2+^ solution (Ca^2+^ free solution with 0.18 mM CaCl_2_). The mid-myocardium from the digested perfused area was cut into small pieces and triturated for 5 min in the low Ca^2+^ solution. Isolated cardiomyocytes were then filtered through a 250 μm mesh and resuspended in low Ca^2+^ solution until use.

### Electrophysiology

Coverslips containing the cells (isolated from 2D hiPSC-CM, 3D constructs, or adult human hearts) were mounted in a chamber perfused with normal Tyrode solution warmed at 37°C and containing (in mM): 137 NaCl, 5.4 KCl, 1.8 CaCl_2_, 0.5 MgCl_2_, 5.5 glucose, and 10 HEPES; pH was adjusted to 7.4 with NaOH. Patch-clamp pipettes (2–3 MΩ) (GB200-8P—Science Products) were filled with a solution containing (in mM): 120 K-Asp, 20 KCl, 10 HEPES, 5 Mg-ATP, 10 NaCl, and 0.05 Fluo-4 (ThermoFisher Scientific—F14200); pH was adjusted to 7.2 with KOH. Cells were patched in a whole-cell configuration, and action potentials were measured using an Axon 200B amplifier and Digidata 1550B (Molecular Device) in current-clamp mode. Stimulated action potentials were recorded after a 5 ms pulse of 0.1 nA at a 1 Hz frequency. To measure voltage-gated calcium currents (ICaL), the setup was set to voltage-clamp mode. A train of seven pulses of 250 ms from −70 to +10 mV was followed by a sodium channel activation pulse of 750 ms from −70 to −40 mV, and then ICaL was recorded with increasing steps of 10 mV of 250 ms from −50 to +60 mV.

### Immunostaining

Snap frozen tissue of adult human hearts embedded in optimal cutting temperature compound (OCT) were cut using a cryostat (Leica) and directly fixed with 4% paraformaldehyde for 10 min (Santacruz Biotechnology—sc-281692). HiPSC-CM in 2D monolayers were directly cultured in imaging plates (Ibidi—82406) and fixed with 4% paraformaldehyde for 15 min. HiPSC-CM in 3D constructs were directly fixed with the silicon posts with 4% paraformaldehyde for 20 min. After fixation, the samples were washed with phosphate-buffered saline (PBS) and permeabilized with 0.4% Triton X-100 (ThermoFisher Scientific—28314) diluted in PBS. Samples were washed three times with PBS and incubated with blocking buffer (4% bovine serum albumin, 0.1% Triton X-100 in PBS) for 1 h at room temperature. Primary antibodies were incubated overnight at 4°C in the blocking buffer: α-sarcoglycan (Leica A-SARC-L-CE, 1:10), β-sarcoglycan (Leica B-SARC-L-CE, 1:10), γ-sarcoglycan (Leica G-SARC-CE, 1:10), δ-sarcoglycan (Leica D-SARC-CE, 1:10), α-dystroglycan (DSHB IIH6 C4-s, 1:10), β-dystroglycan [DSHB MANDAG2(7D11)-s, 1:10], dystrophin (Leica DYS1-CE, 1:10), cTnT (Abcam ab92546, 1:200), and α-actinin (Proteintech 14221-1-AP, 1:200). The next day, after three washes in PBS, samples were incubated with secondary antibodies for 2 h at room temperature in the blocking buffer: goat anti-mouse IgG Alexa 488 (ThermoFisher Scientific—A-21121, 1:200) and goat anti-rabbit IgG Alexa 568 (ThermoFisher Scientific—A-11036, 1:200), according to the primary antibody. The sections from the human hearts were mounted using ProLong^TM^ Gold Antifade Mountant with diamino-2-phenyl-indole (DAPI) (ThermoFisher Scientific—P36931). For imaging, the 3D constructs were directly placed on a coverslip. Imaging was performed using a confocal microscope (Nikon A1R configured on an Eclipse Ti2 using a ×60 1.4 NA oil immersion objective).

### Immunoblot

Adult human heart samples and 3D cultured hiPSC-CM were snap frozen in liquid nitrogen and stored at −80°C until use. Homogenization of samples was done on ice using a tissue grinder (Weathon) with the following solution: 10 mM Tris–HCl pH 7.5, 100 mM NaCl, 1 mM EDTA, 1 mM Na_3_VO_4_, 1% sodium deoxycholate, 1% Triton X-100, 1% NP-40, 0.1% sodium dodecyl sulfate (SDS), 10 mM NaF, 1 mM phenylmethylsulfonyl fluoride (PMSF), and protease inhibitor tablets (ThermoFisher Scientific—A32963). Protein concentration was estimated using the bicinchoninic acid (BCA) assay from ThermoFisher Scientific (23225), and aliquots were stored at −80°C until use. For de-glycosylation of proteins, a PNGase kit was used (New England BioLabs—NEB P0704S). Homogenized samples (30 μg) were loaded in a home-made Tris-acetate 3–15% gel, as described (Cubillos-Rojas et al., 2012). After an overnight liquid transfer (4°C at 40 V for 19 h) of the gel to a polyvinylidene difluoride (PVDF) membrane, the membrane was saturated for 45 min with 4% non-fat dry milk (Bio-Rad—1706404) diluted in PBS (pH = 7.4) with 0.05% Tween-20. The membrane was cut at around the 160 kDa marker into two pieces. The top part was used to probe for dystrophin and the lower part for sarcoglycans and dystroglycans (Supplementary Figure 3). Then, membranes were incubated overnight at 4°C with the primary antibodies diluted in 2% milk (same antibodies as used for immunostainings, 1:1,000 dilution). The next day, after three washes in PBS, membranes were incubated for 2 h at room temperature with secondary antibodies: goat anti-mouse IgG Alexa 680 (1:10,000, ThermoFisher Scientific—A28183). Membrane immunofluorescence was quantified with a Licor Odyssey Clx infrared imaging system.

### Polymerase Chain Reaction

Adult human heart samples and 3D-cultured hiPSC-CM were snap frozen in liquid nitrogen and stored at −80°C until use. Homogenization of samples was done using ceramide beads (MP Biomedicals—116913050-CF) in 1 ml of TRI Reagent (Merk—93289) and using the MP Biomedical Instrument FastPrep-24 grinder (MP Biomedicals) at a speed of 6 m/s for 20 s, twice. Chloroform (0.2 ml) was added per milliliter of TRI Reagent and incubated for 3 min at room temperature. After centrifugation at 12,000 *g* for 15 min at 4°C, the upper phase containing RNA was collected. To this, 0.5 ml of isopropanol per milliliter of TRI Reagent was then added and incubated for 5 min at room temperature. Samples were then centrifuged at 12,000 *g* for 10 min at 4°C and the supernatant removed. The pellet was then washed with 1 ml of ethanol 75% and centrifuged at 7,600 *g* for 5 min at 4°C and the supernatant discarded. The RNA pellet was resuspended in 20 μl of DNase/RNase-free water. cDNA was generated from the RNA extracted samples by reverse transcription using a kit (ThermoFisher Scientific—4368814). The cDNA was then polymerase chain reaction (PCR) amplified using the Platinum^®^ Taq DNA Polymerase High Fidelity kit (ThermoFisher Scientific—11304011) with the following primers: α-SG (TGAGGTCACAGCCTACAATCG and AACTCGGCTTGGTATGGCAG), β-SG (AGCAAAGT TCCAATGGTCCTG and TCATCAATCGGAATGTATCCAGC), γ-SG (GAGCAGTACACTACAGCCACA and CGCAGTCCA TCTTTTGTTACACA), and δ-SG (GCGGAAACGATGCCT GTATTT and TGGCGTAGAGAGGTTGTAAGAA). The PCR products were resolved on a 2% agarose gel for 30 min at 50 V using SYBR^®^ Safe DNA Gel Stain (ThermoFisher Scientific—S33102) and visualized with UV light exposure using a GelDoc Imaging System (Bio-Rad). For RT-qPCR, Platinum^TM^ SYBR^TM^ Green qPCR SuperMix-UDG was used (ThermoFisher Scientific—11733038) and run on a ViiA 7 Real-Time PCR System (ThermoFisher Scientific). The gene expression was normalized to housekeeping genes (GAPDH and RLP13a), and values were expressed as 2^–ΔΔCT^ as a fold difference to adult.

### Statistics

Graphs were prepared and data analyzed using GraphPad Prism software version 9. Normality was tested with Shapiro–Wilk. Except for resting membrane potential, the data did not pass the normality test, and hence groups were compared using Kruskal–Wallis with Dunn’s multiple comparison. For the analysis of the resting membrane potential, we used Welch ANOVA, with Dunnett T3 for multiple comparisons. *P*-values are indicated above each graph. Individual data points are displayed in the graphs with the mean and the standard error of the mean as error bars.

## Results and Discussion

**FIGURE 2:**
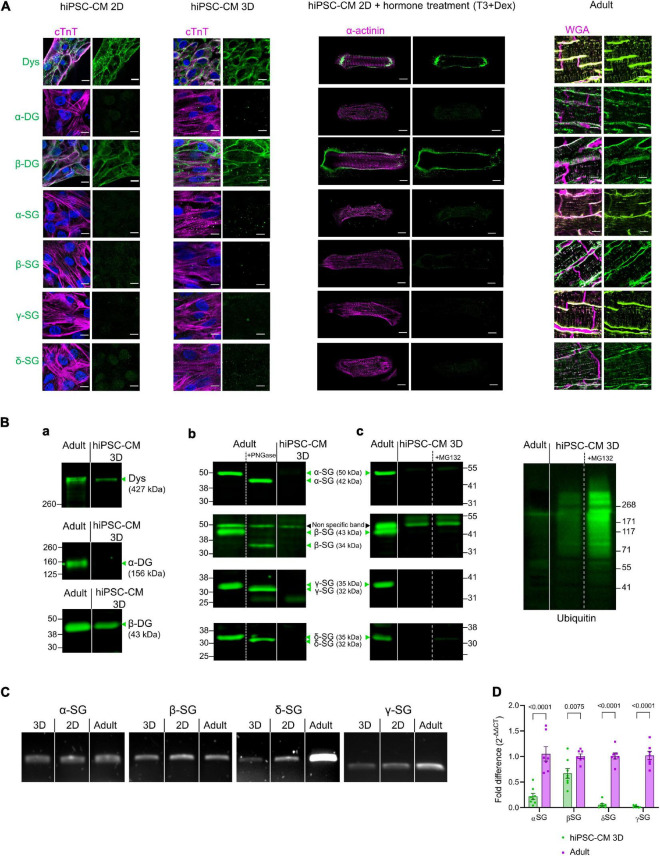
**(A)** From left to right: confocal images of immunostained 2D monolayer cultured hiPSC-CM, 3D-cultured hiPSC-CM tissue, 2D-cultured hiPSC-CM seeded on Matrigel mattresses, and treated with T3+Dexamethasone and cryosections of adult human heart tissue. Adult heart sections were counterstained with wheat germ agglutinin (WGA) for membrane, shown in magenta. hiPSC-CM were counterstained with cardiac troponin T (cTnT) or α-actinin, shown in magenta. Nuclei were labeled with DAPI in blue. The DAPC components are in green. Results were replicated in three independent hiPSC-CM differentiations. Scale bar, 10 μm. **(B)** **(a)** Immunoblot of dystrophin (Dys) and dystroglycan (DG). **(b)** Immunoblot of sarcoglycans (SG) and Deglycosylation tests (+PNGase treatment) in adult cardiac homogenates and in 3D-cultured hiPSC-CM. Results were replicated in seven independent hiPSC-CM differentiations for α-SG, δ-SG, and γ-SG; 10 independent differentiations for β-SG and dystrophin and 5 independent differentiations for dystroglycan (DG). **(c)** Immunoblot for sarcoglycans from 3D-cultured hiPSC-CM treated for 8 h with MG-132 (10 μM). The proteasome inhibition efficiency was confirmed by immunoblotting of lysates with an anti-ubiquitin antibody (right). Results are from four 3D constructs prepared from one hiPSC-CM differentiation. Straight lines separate adult vs. hiPSC-CM, and dotted lines separate control vs. treatment (PNGase or MG132), from the same blot. The expected molecular weight for glycosylated and deglycosylated forms of sarcoglycans are indicated in the figure. The β-SG band at 50 kDa was considered as non-specific as its molecular weight did not decrease with deglycosylation (a similar band was also seen in β-SG-null mouse heart—Supplementary Figure 2B). **(C)** mRNA detection by reverse transcription PCR of expression of sarcoglycans in 2D and 3D-cultured hiPSC-CM and in adult human heart lysates. Results were replicated in three independent hiPSC-CM differentiations. **(D)** RT-qPCR of sarcoglycans in 3D-cultured hiPSC-CM and adult human heart lysates. Values are expressed as 2^–ΔΔCT^ normalized as a fold difference to adult.

In adult cardiomyocytes, the core proteins of the DAPC [dystrophin, dystroglycans (α and β), and sarcoglycans (α, β, γ, and δ)] were present at the membrane, both in the external plasmalemma and in transverse tubules but not at the intercalated discs (Figure 2A, right). In contrast, hiPSC-CM generated using a common 2D monolayer protocol expressed an incomplete DAPC with only dystrophin and β-dystroglycan present (Figure 2A, left). We investigated whether a further maturation process could improve the expression of the proteins that comprise the DAPC, especially sarcoglycans as important mediators of dystrophy-related cardiomyopathies. To these ends, two protocols were used: the first in which we cultured hiPSC-CM in a 3D microenvironment and a second in which we combined a treatment with triiodo-L-thyronine and glucocorticoid for 14 days before seeding the cells on 2D Matrigel mattresses. However, neither maturation protocol improved DAPC expression above that seen in 2D hiPSC-CM, which only express dystrophin and β-dystroglycan but not sarcoglycans or α-dystroglycan (Figure 2A, middle). These findings were confirmed in immunoblots in the 3D-cultured hiPSC-CM (Figure 2B, panel a and b). All data presented here are from the ThermoFisher Scientific hiPSC line, and similar data were obtained in 3D cultures from three different hiPSC lines, one from the Leuven and two from the Hamburg Labs (Supplementary Figures 1,2). The specificity of the sarcoglycan antibodies used was further verified by deglycosylating the proteins in adult cardiac homogenates with PNGase F, and as expected, all sarcoglycans decreased in molecular weight after deglycosylation (Figure 2B, panel b). Inhibition of the proteasome by treatment with MG-132 (10 μM) for 8 h to reduce protein degradation had no effect and could not uncover sarcoglycan expression (Figure 2B, panel c). Yet, hiPSC-CM expressed sarcoglycans at the mRNA level (Figure 2C). Additional RT-qPCR experiments showed differences in expression of components of the DAPC between 3D culture hiPSC-CM and adult cardiac tissue (Figure 2D).

**FIGURE 3:**
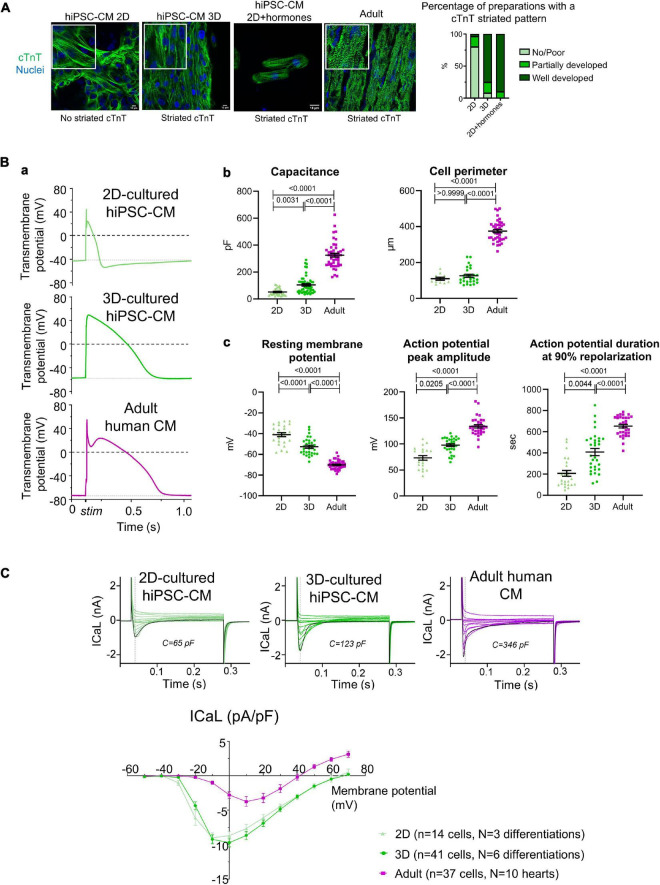
**(A)** Cardiac troponin T (cTnT) organization in hiPSC-CM. Left: immunostaining for cTnT (green) and nuclei (blue) in hiPSC-CM cultured in 2D, 3D, or 2D+hormones seeded on Matrigel mattresses, and for comparison in adult human cardiac tissue. Right: semi-quantitative analysis of the cTnT striated pattern (2D: *n* = 25 preparation from five independent differentiations; 3D: *n* = 24 preparations from six independent differentiations; 2D+hormones: *n* = 10 preparations from two independent differentiations). **(B)** **(a)** Representative example of electrically stimulated action potentials. **(b)** Analysis of cell capacitance (2D-hiPSC-CM: *n* = 34 cells; 3D-hiPSC-CM: *n* = 52 cells; adult cardiomyocytes: *n* = 46 cells) and cell perimeter (2D-hiPSC-CM: *n* = 11 cells; 3D-hiPSC-CM: *n* = 27 cells; adult cardiomyocytes: *n* = 39 cells). **(c)** Analysis of resting membrane potential (2D-hiPSC-CM: *n* = 25 cells; 3D-hiPSC-CM: *n* = 33 cells; adult cardiomyocytes: *n* = 43 cells), action potential amplitude (2D-hiPSC-CM: *n* = 21 cells; 3D-hiPSC-CM: *n* = 31 cells; adult cardiomyocytes: *n* = 32 cells), and duration at 90% repolarization (2D-hiPSC-CM: *n* = 21 cells, *N* = 3 differentiations; 3D-hiPSC-CM: *n* = 31 cells, *N* = 6 differentiations; adult cardiomyocytes: *n* = 32 cells, *N* = 10 hearts). **(C)** Top: representative example of ICaL over membrane potentials spanning from –40 to +70 mV. The black curve represents the voltage step at 0 mV. Bottom: current-voltage curve of the ICaL measured at the peak current (*n* numbers are indicated in the graph). *P*-values are indicated above each graph; Kruskal–Wallis with a Dunn’s multiple comparison test was used for all, except for resting membrane potential where a Welch ANOVA test, followed by Dunnett T3 for multiple comparisons, was used.

We examined proxies for maturation in the present experiments, focusing on aspects of excitation–contraction coupling as a key feature of cardiomyocytes. The increased sarcomeric organization in hiPSC-CM cultured in 3D and with hormonal treatment in 2D supported the assumption of advanced maturation of the myocyte phenotype under these conditions (Figure 3A). We also evaluated how 3D culture influences the electrophysiological properties of hiPSC-CM, compared to cells cultured in 2D monolayers and to adult human ventricular cardiomyocytes. Figure 3B, panel a shows representative examples of single cell action potentials. Both hiPSC-CM cultured in 2D and 3D were smaller than adult ventricular cells, in cell perimeter and electrical capacitance, though the latter was higher in 3D-cultured hiPSC-CM (Figure 3B, panel b). In addition, compared to 2D-cultured cells, 3D-cultured hiPSC-CM had a more negative resting membrane potential and greater action potential amplitude and duration, with values closer to that in adult ventricular cardiomyocytes (Figure 3B, panel c). Considering we did not correct for junction potentials, these values for resting membrane potential are comparable to those previously reported (Horvath et al., 2018). Of note, the resting membrane potential measured with microelectrodes in hiPSC-CM within the connected 3D micro-tissue are more negative than those after isolation (Horvath et al., 2018). These features seen in 3D culture (lower resting membrane potential and longer action potential duration) are considered a characteristic of a more adult and ventricular-like cardiomyocyte phenotype. Compared to adult cardiomyocytes, both 2D and 3D-cultured hiPSC-CM had a higher density of L-type voltage-gated calcium channel current (ICaL) (Figure 3C), probably related to the absence of T-tubules and consequent smaller membrane surface area. Adult cardiomyocytes typically have a fast inactivation phase of ICaL caused by Ca^2+^ release from the internal store, followed by a slow phase. ICaL in 2D-cultured hiPSC-CM typically has a single inactivation phase, while ICaL in 3D-cultured hiPSC-CM can have either type of inactivation time course. These data highlight that the link between calcium influx and sarcoplasmic reticulum release of calcium may improve in 3D but remains poorly developed.

Taken together, our findings show that despite evidence for a more advanced maturation, 3D-cultured-hiPSC-CM lack the complete DAPC seen in adult cardiomyocytes: dystrophin and β-dystroglycan are present, but sarcoglycans and α-dystroglycan are not (Figure 1B). The lack of a full DAPC in hiPSC-CM, even after additional culture in 3D or with hormonal treatment, may reflect the incomplete maturity of the cells. Interestingly, during the early stages of human fetal development, the heart expresses sarcoglycans at the mRNA but not at the protein level, only expressing dystrophin and β-dystroglycan (Mora et al., 1996; Fougerousse et al., 1998), and this is in line with the immaturity or “fetal-like” phenotype of hiPSC-CM. The expression of dystrophin in hiPSC-CM, even already present in 2D monolayer differentiated cells, confirms the use of hiPSC-CM to study Duchenne muscular dystrophy. However, the lack of sarcoglycans undermines the use of hiPSC-CM as a model for sarcoglycanopathies and suggests caution in the interpretation of the dystrophin studies. The absence of α-dystroglycan, as recently observed (Kamdar et al., 2020), would prevent the linking of the complex to laminin and the extracellular matrix, thereby potentially affecting mechanosensing signaling, which is important for cell adaptation and maturation. However, we cannot rule out that we did not detect α-dystroglycan in our samples due to its release into the culture medium, as this extracellular protein could be poorly retained in an immature DAPC. It is conceivable that the incomplete DAPC is one of the hurdles to progression to an adult phenotype of hiPSC-CM. Recent protocols using co-cultures with fibroblasts and endothelial cells may further improve maturation but, because of their complexity, are not yet widely adopted (Giacomelli et al., 2020).

## Conclusion

In conclusion, because of the unique insight into patient-specific genetic and functional background they provide, hiPSC-CM are a highly relevant model to study genetic cardiac diseases. However, our findings indicate that it is important to recognize the limitations of the hiPSC-CM model for the study of dystrophy-related cardiomyopathies. Further understanding of the mechanisms that govern the stabilization of sarcoglycans and α-dystroglycan within the DAPC can improve the use of hiPSC-CM as a model system and as a bridge to medical applications such as regenerative medicine and drug screening.

## Data Availability Statement

The original contributions presented in the study are included in the article/Supplementary Material, further inquiries can be directed to the corresponding authors.

## Ethics Statement

The studies involving human participants were reviewed and approved by the Ethical Committee of UZ Leuven (permit number S58824). Written informed consent for participation was not required for this study in accordance with the national legislation and the institutional requirements.

## Author Contributions

GG, MS, and KS: conceptualization. GG and KS: formal analysis, visualization, and writing—original draft. GG, KS, HLR, MS, and RD: funding acquisition. GG, CK, RD, and MA: investigation. GG, RD, CK, PB, and TE: methodology. KS and MS: project administration. KS, MS, HLR, and TE: resources. GG: software. KS, MS, and HR: supervision. KS: validation. All authors contributed to the writing—review and editing.

## Conflict of Interest

TE is co-founder of EHT Technologies GmbH, Hamburg. The remaining authors declare that the research was conducted in the absence of any commercial or financial relationships that could be construed as a potential conflict of interest.

## Publisher’s Note

All claims expressed in this article are solely those of the authors and do not necessarily represent those of their affiliated organizations, or those of the publisher, the editors and the reviewers. Any product that may be evaluated in this article, or claim that may be made by its manufacturer, is not guaranteed or endorsed by the publisher.

## Abbreviations

DAPC: dystrophin-associated protein complex
CM: cardiomyocyte
DG: dystroglycan
hiPSC-CM: human induced pluripotent stem cell-derived cardiomyocytes
SG: sarcoglycan.
