# Supplementary material for: Incomplete Assembly of the Dystrophin-Associated Protein Complex in 2D and 3D-Cultured Human Induced Pluripotent Stem Cell-Derived Cardiomyocytes
Source: Front Cell Dev Biol. 2021 Nov 4;9:737840. doi: 10.3389/fcell.2021.737840 (PMC8599983; doi:10.3389/fcell.2021.737840)
Supplement: Supplementary file 1 [file Data_Sheet_1.PDF]

Supplementary Figure 1 - Immunostaining on HC1 cell line

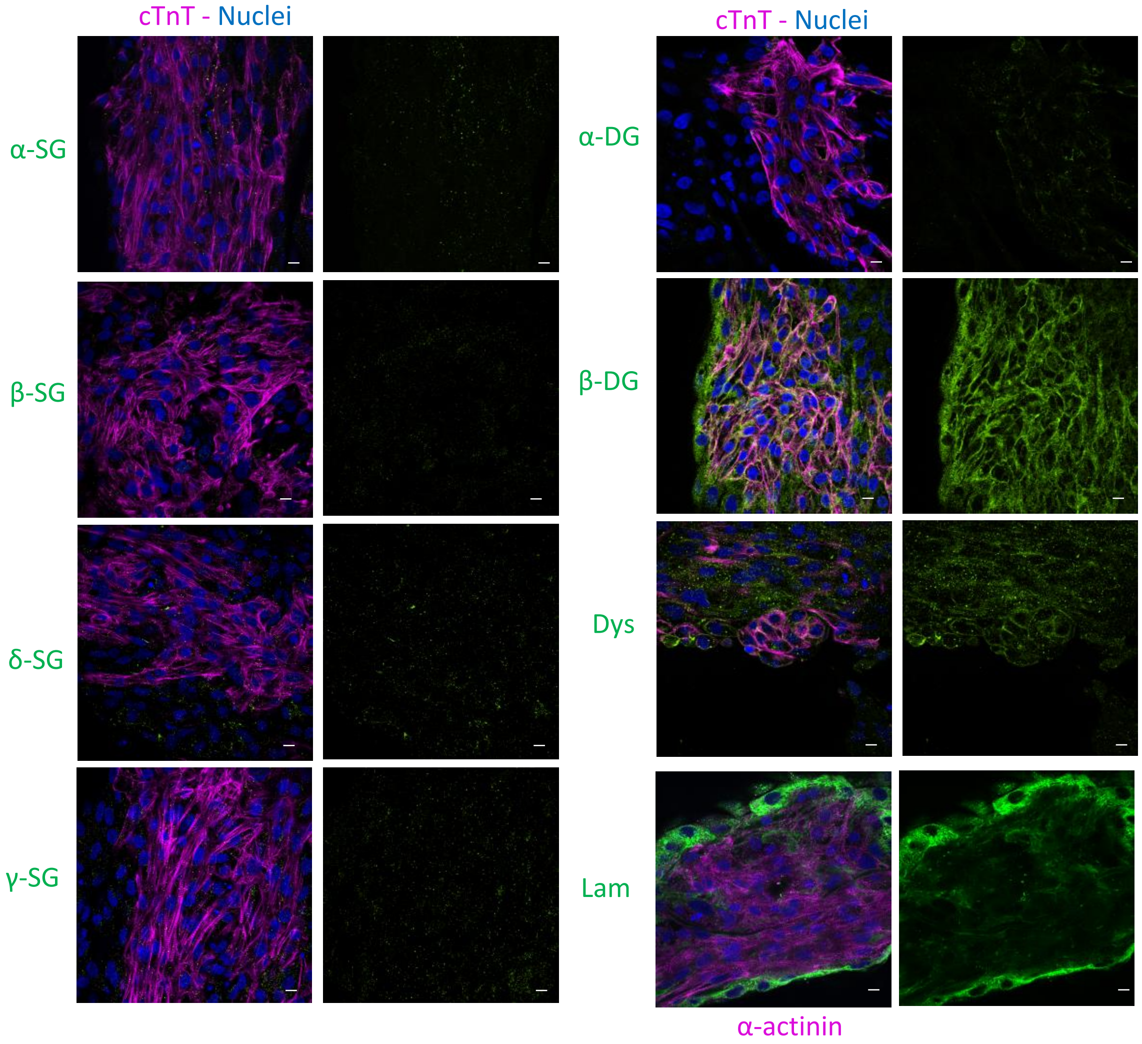

Immunostaining of 3D-cultured hiPSC-CM from the HC1 cell line for the different proteins of the DAPC (in green) and cytoskeleton (cTnT or alpha-actinin) in purple. Image were taken in a NIKON A1R confocal microscope, oil immersive 60x objective, NA 1.4. We observe that the cells do not express α/β/γ/δ sarcoglycans and α-dystroglycan but express dystrophin, beta-dystroglycan and laminin. Scale bar = 10 µm.

Supplementary Figure 2 – Immunoblot on HC1, ERC001 and ERC018 cell lines

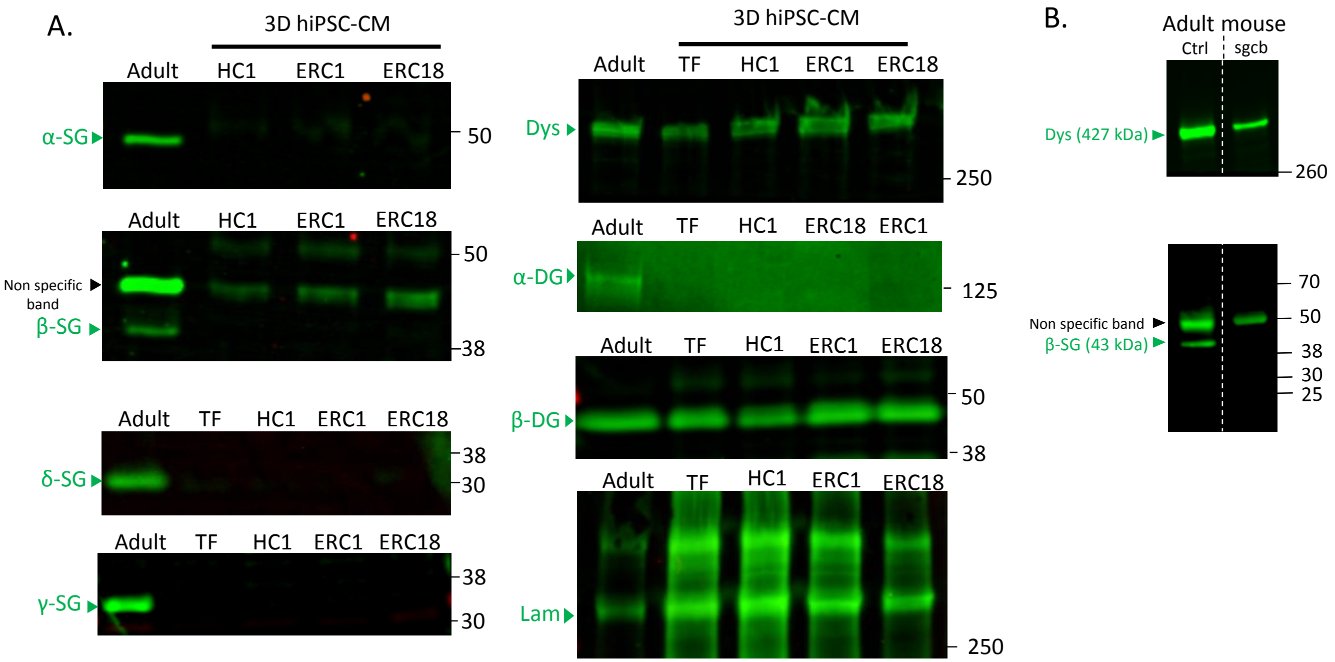

A. Immunoblot for the different proteins of the DAPC (sarcoglycans (SG), dystrophin (Dys), dystroglycans (Dys) and laminin (Lam), on adult heart tissue (Adult), used as a positive control and on 3D-cultured hiPSC-CM cell lines: TF (ThermoFisher cell line), HC1 (Leuven), ERC1 and ERC18 (Hamburg lab). Arrows on the left side of the blots indicate the size of the proteins of interest. Molecular weights on right side of the blots are in kDa. We observe that the 4 cell lines of hiPSC-CM express dystrophin, beta-dystroglycan and laminin but no sarcoglycans nor alpha-dystroglycan. The band below 50 kDa for beta-sarcoglycan observed in hiPSC-CM and adult tissue was considered as non-specific because too high for beta-sarcoglycan compared to the band observed in adult tissue (at 43 kDa, indicated by the arrow on the left side) and because we still observed this non-specific band in beta-sarcoglycan null mice (sgcb) heart samples (**B**).

Supplementary Figure 3 – Full western blot with dystrophin on top and sarcoglycans on the bottom part

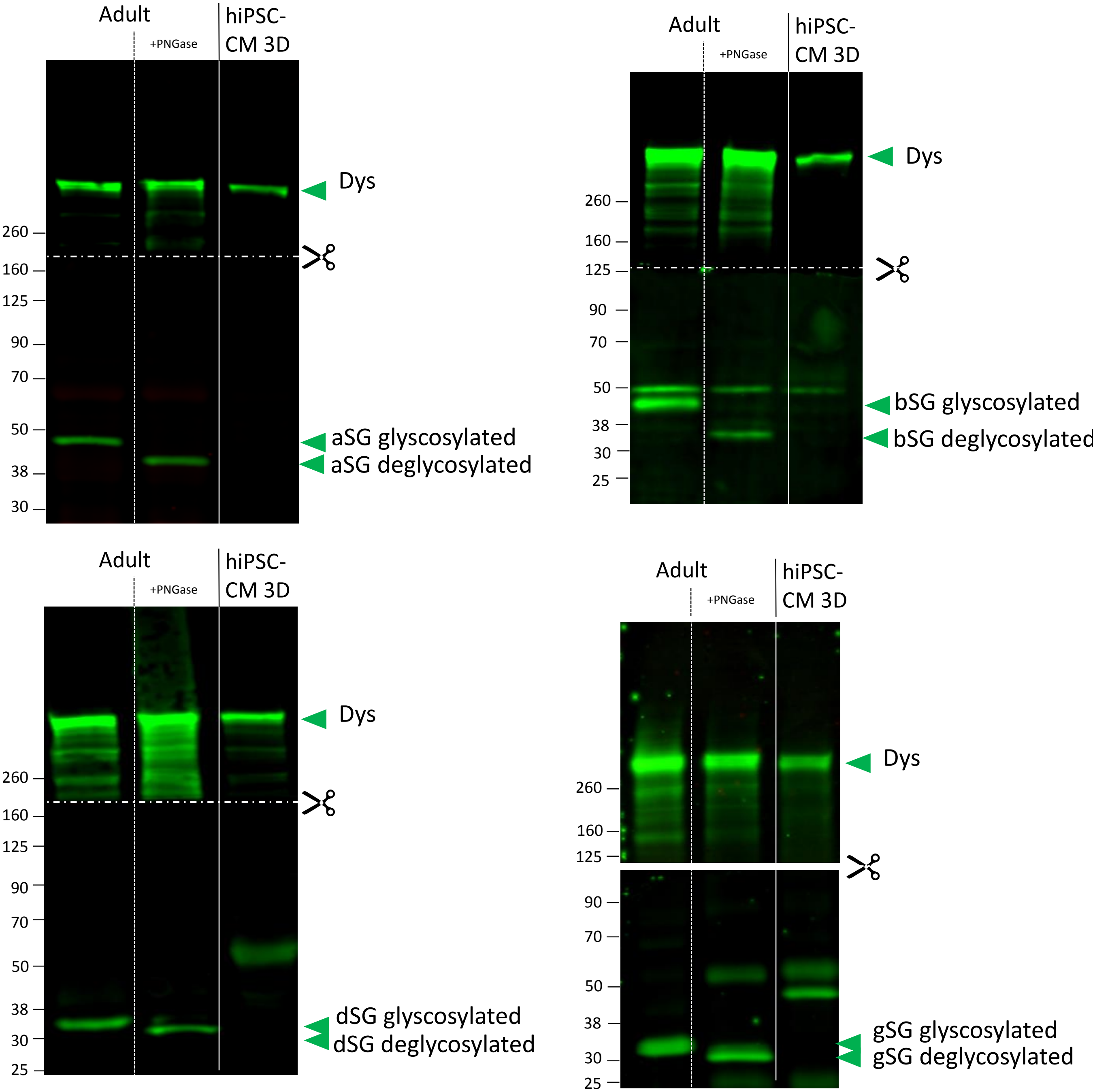

Immunoblot for dystrophin (upper part) and sarcoglycans (SG) (lower part) performed on adult human heart tissue (control and treated with PNGase) and on 3D-cultured hiPSC-CM. The bands for glycosylated and deglycosylated forms of sarcoglycans, observed in adult samples are indicated with the arrows.

Supplementary Figure 4 – Electrophysiology data on 2D+hormone treated hiPSC-CM

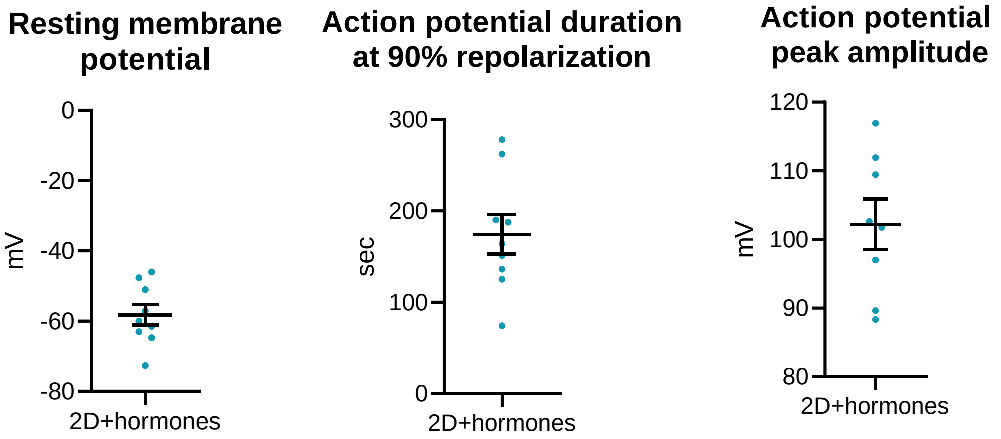

Resting membrane potential, ADP90 and action potential amplitude in patched cells dissociated from hiPSC-CM cultured in 2D with hormones (thyroid hormone and glucocorticoids) and seeded on matrigel mattresses.
